# Exploring the relationship between the level of facial nerve injury and surgical outcome: Application of a new anatomical classification system in a limited cohort undergoing marginal mandibular nerve reconstruction

**DOI:** 10.1016/j.jpra.2025.01.013

**Published:** 2025-01-25

**Authors:** Villiam Vejbrink Kildal, Alex Okello Wamalwa, Ludvig Tidehag Walan, Andrés Rodriguez-Lorenzo

**Affiliations:** a Department of Surgical Sciences, Plastic and Maxillofacial Surgery, Uppsala University, Uppsala, Sweden; b Department of Plastic and Maxillofacial Surgery, Uppsala University Hospital, Uppsala, Sweden; c Department of Clinical Science and Education, Section of Anesthesiology and Intensive Care, Södersjukhuset, Karolinska Institutet, Stockholm, Sweden; d Department of Anesthesiology and Intensive Care, Södersjukhuset, Stockholm, Sweden; e Department of Otolaryngology, Uppsala University Hospital, Uppsala, Sweden

**Keywords:** Lower lip reanimation, Marginal mandibular nerve reconstruction, Nerve graft, Nerve transfer, Nerve repair, Facial paralysis

## Abstract

**Background:**

Reconstruction of the marginal mandibular nerve (MMN) is important for achieving optimal outcomes in the treatment of facial paralysis. However, the heterogeneity of injuries, ranging from extensive proximal facial nerve injuries to isolated distal MMN injuries, complicates meaningful outcome comparisons. This study assessed a new anatomical classification system for stratifying facial nerve injuries by injury location. The aim was to study MMN outcomes in proximal versus distal injuries and determine whether this system could provide a more reliable way to compare surgical results.

**Methods:**

A retrospective, single-center study of MMN reconstructions (either independent MMN reconstructions or as part of a broader facial nerve reconstruction) was conducted over a 12-year period. Clinical outcomes were assessed using a classification system for facial nerve injuries (Levels 1−3, based on facial nerve injury location: Level 1 = proximal, Level 2 = parotid area, Level 3 = distal). Outcome measures included the Terzis' Lower Lip Grading Scale, photogrammetry, Sunnybrook, and quality-of-life assessments (Facial Disability Index, Facial Clinimetric Evaluation Scale).

**Results:**

Sixteen patients (7 female; mean age 46.5 ± 20.6 years) underwent MMN reconstruction. Across all outcome measures, distal Level 3 injuries yielded the best outcomes, followed by Level 2, with proximal Level 1 injuries showing the least favourable results.

**Conclusions:**

Proximal facial nerve injuries demonstrated inferior MMN outcomes compared with distal injuries, highlighting the importance of considering injury location when comparing results. The proposed classification system may provide a practical method for grouping patients according to anatomical injury location, enabling more meaningful and standardized comparisons of surgical outcomes among patients with similar characteristics and treatment protocols.

## Introduction

Facial paralysis affecting the lower face can lead to significant functional and aesthetic impairments, as the mouth and lips play a vital role in shaping facial expression and overall identity.^1^, ^2^, ^3^, ^4^, ^5^, ^6^ The mobility of the lower lip is primarily dependent on the depressor anguli oris, depressor labii inferioris, and mentalis muscles, all of which receive part of their motor innervation from the marginal mandibular nerve (MMN).^5^ MMN paralysis may therefore result in an incompetent lip, characterized by the conspicuous presentation of a weak, flat, and inverted lower lip that is unable to move inferiorly and laterally, and cannot pout, which can significantly impair both physiological and social functioning.^6^ Therefore, restoring the function of the lower lip depressor muscles through dynamic nerve reconstruction is necessary to achieve optimal outcomes in the treatment of facial paralysis.^5^^,^^7^, ^8^, ^9^

Surgical outcomes are often compared based on the method of reconstruction.^5^^,^^6^^,^^10^, ^11^, ^12^, ^13^ However, a key issue is that patients undergoing MMN repair represent a highly heterogeneous group, comprising both extensive proximal facial nerve injuries and isolated distal MMN injuries, which complicates meaningful comparisons of outcomes. Patients undergoing MMN repair using the same general reconstruction method (e.g., nerve graft) may experience vastly different outcomes depending on factors such as etiology, nerve gap length, possible timing of reconstruction, and the potential for effective rehabilitation. Thus, comparing outcomes solely based on the surgical method risks combining disparate clinical situations, which might be misleading and could complicate the ability to draw meaningful conclusions. By contrast, a classification system based on anatomical injury location could provide a more standardized approach for categorizing patients with different types of facial paralysis into more uniform groups, enabling more meaningful comparisons of surgical outcomes by matching patients with similar injury and reconstruction characteristics. Injury location determines several important variables that influence outcomes. For example, in patients with proximal facial nerve injuries and complete unilateral facial paralysis, MMN reconstruction can yield poorer outcomes due to often more complex reconstructions, longer nerve gaps, and a greater emphasis on postoperative rehabilitation targeting the eye and upper smile rather than lower lip movement. By contrast, distal MMN injuries typically involve shorter nerve gaps and less extensive damage to the facial nerve, enabling simpler reconstructions with better outcomes.

This study aimed to stratify patients undergoing MMN reconstruction using a new, simple, anatomical classification system for facial nerve injuries based on injury location. The primary goal was to evaluate how proximal versus distal facial nerve injuries, with their differing clinical scenarios and available treatment alternatives, influence MMN outcomes. A secondary aim was to determine whether this classification system could group patients with similar characteristics, facilitating more meaningful and standardized comparisons of surgical outcomes.

## Methods

The study was performed in accordance with the local rules and regulations and was approved by the ethical institutional committee in Sweden (Dnr 2020-03492 with amendments 2022-04681-02 and 2023-01156-02). All patients signed letters of consent for participation in the study and for publication of photographs and/or video materials.

**Figure 1:**
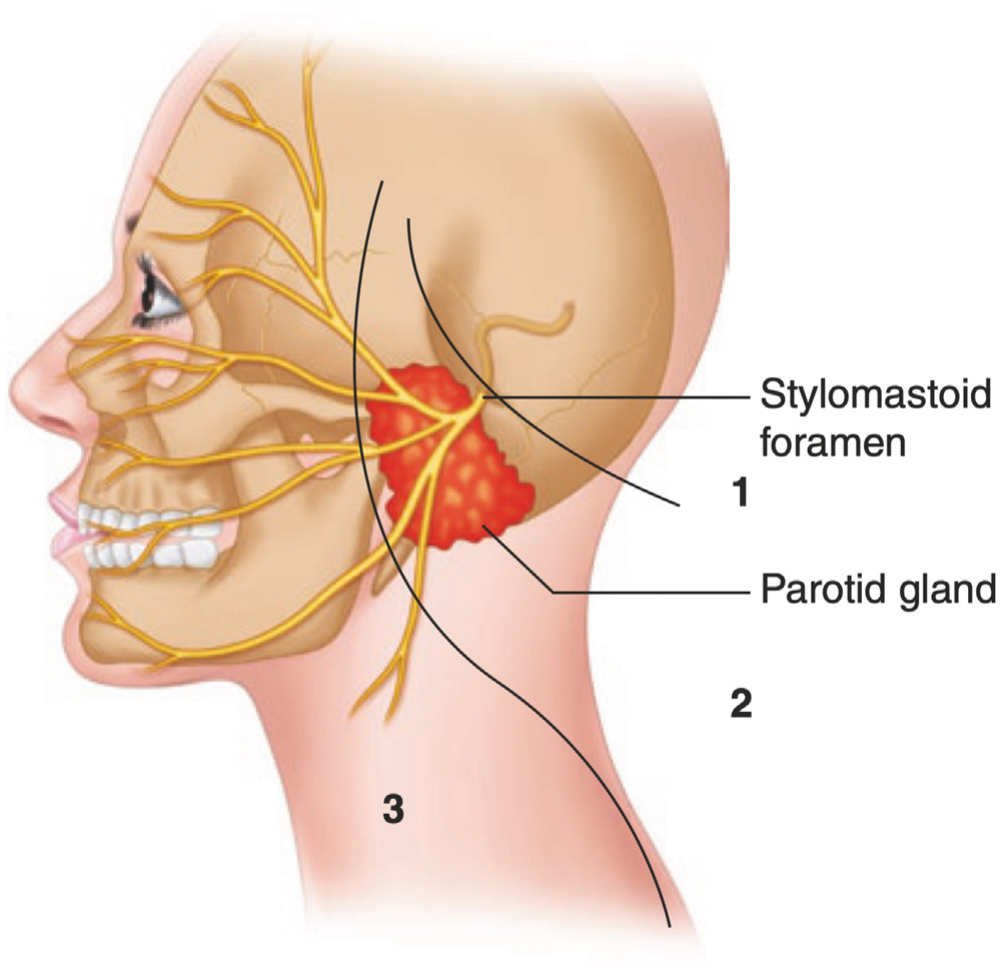
Classification of facial nerve injury levels: Level 1: Intracranial or intratemporal nerve injuries with no available proximal facial nerve stump; Level 2: Intratemporal or extratemporal/intraparotid injuries with the proximal facial nerve stump available; Level 3: Extratemporal extraparotid nerve injuries with the proximal facial nerve stump available. Illustration borrowed from the book “Facial Palsy: Techniques for Reanimation of the Paralyzed Face” with permission from the publisher.^23^

This retrospective single-center case study included patients who underwent MMN reconstruction between January 2010 and September 2022 at the Department of Plastic and Maxillofacial Surgery, Uppsala University Hospital, Sweden. The following information was extracted from the patient records: age, sex, comorbidities, etiology of facial paralysis, surgical method of nerve reconstruction, the time between injury and reconstructive surgery, potential secondary surgery, time from reinnervation surgery to first signs of reanimation, the total length of follow-up, Sunnybrook^14^ scores, and potential complications graded according to the Clavien-Dindo scale.^15^ The level of facial nerve injury was determined according to a novel classification system previously proposed by the senior author.^15^ The levels are demonstrated in Figure 1 and is defined as follows: Level 1 involves intracranial or intratemporal facial nerve injuries, where the proximal facial nerve stump is unavailable; Level 2 encompasses intratemporal or intraparotid facial nerve injuries, with the proximal stump available; and Level 3 pertains to injuries distal to the parotid gland, where the proximal stump is available.

The following outcome parameters were analyzed according to the level of facial nerve injury (see the definition above). Photographs and videos from the latest available follow-up were extracted and analyzed using photogrammetry (Emotrics^16^). The results are presented as the mean of the absolute parameters of commissure excursion, commissure height, smile angle deviation, upper lip height deviation, dental show, and lower lip height deviation. The modified Terzis’ Lower Lip Grading Scale^11^^,^^17^ (a scale from 1 [total lower lip paralysis] to 5 [normal symmetric movement]) graded by 14 independent observers (doctors from the Department of Plastic and Maxillofacial Surgery and the Department of Otorhinolaryngology Surgery) for subjective grading of the lower lip function. The Sunnybrook system was utilized, and patients were asked to fill out questionnaires for symptom grading and quality-of-life (Facial Clinimetric Evaluation scale^18^^,^^19^ [FaCE scale] and Facial Disability Index^19^^,^^20^ [FDI]).

### Statistical analysis

All data processing, statistical analysis, and graphical representations were performed using R statistical computing software (version 4.3.0 [2023-04-21])^21^ in the Tidyverse ecosystem.^22^ Assumptions of normality and homoscedasticity were tested on the residuals using quantile-quantile plots and Levene's test. We used the non-parametric Kruskal-Wallis test to examine all variables because the cohort was small and some were not homoscedastic. We also used Dunn-Bonferroni post-hoc tests to compare pairs of subgroups. All tests were two-sided, with a significance level of α = 0.05. To quantify the magnitude of the observed differences, effect sizes were calculated using eta-squared (η^2^) based on the Kruskal-Wallis H-statistic, where commonly interpreted values are between 0.01–0.06 (small effect), 0.06–0.14 (moderate effect), and >0.14 (large effect).

## Results

Eighteen patients underwent MMN reconstruction performed by the same surgeon within the designated timeframe. Two patients were excluded due to death before the first follow-up, resulting in a lack of photos and videos for analysis. Sixteen patients were included (7 females; mean age, 46.5 ± 20.6 [range 6–71]). In 2 of these patients, quality-of-life scores could not be obtained; 1 patient died before the questionnaires could be answered, and another experienced a tumor recurrence and renewed facial paralysis after the initial reconstruction and thus had a reduced quality-of-life unrelated to the initial MMN reconstruction.

The level of facial nerve injury was Level 1 in 5 patients, Level 2 in 7, and Level 3 in 4. All nerve reconstructions were performed under an operating microscope using nonabsorbable monofilament (9.0) sutures and fibrin glue. The surgical methods are listed in Table 1 together with further descriptive statistics.

**Table 1:** Descriptive statistics of the included patients.

Descriptive statistics				
Summary statistics by level of injury				
Level of injury	All levels	1	2	3
Number of patients	16	5	7	4
Female	7	4	1	2
Right-sided paralysis	7	2	3	2
No. complications	10	3	4	3
Length of follow-up ± SD (mo)	31 ± 16	33 ± 17	31 ± 16	29 ± 19
Age ± SD (y)	46 ± 21	44 ± 19	53 ± 16	39 ± 31
Etiology				
Tumor	13	4	7	2
Trauma	2	0	0	2
Structural	1	1	0	0
Surgical method				
Direct nerve repair	1	0	0	1
Cervical branch	4	1	0	3
Ansa cervicalis	4	0	4	0
Hypoglossal	6	4	2	0
Nerve graft	1	0	1	0

“All Levels” include the full cohort (all patients from Levels 1, 2, and 3), and then each level is presented separately. Percentages should be viewed vertically.

For example, the group “All Levels” should be interpreted as follows: in the group, 44% of the patients were female, the average age was 46 ± 21 years, and of the etiologies in that group, 81% had tumors, 12% had trauma, and 6% had structural causes.

All nerve transfers were performed using a direct end-to-end method, except for 1 case of hypoglossal transfer, where an interposition nerve graft was necessary to achieve the required reach, and 2 cases of cervical nerve transfer, where a nerve graft from the contralateral side was used.

**Figure 2:**
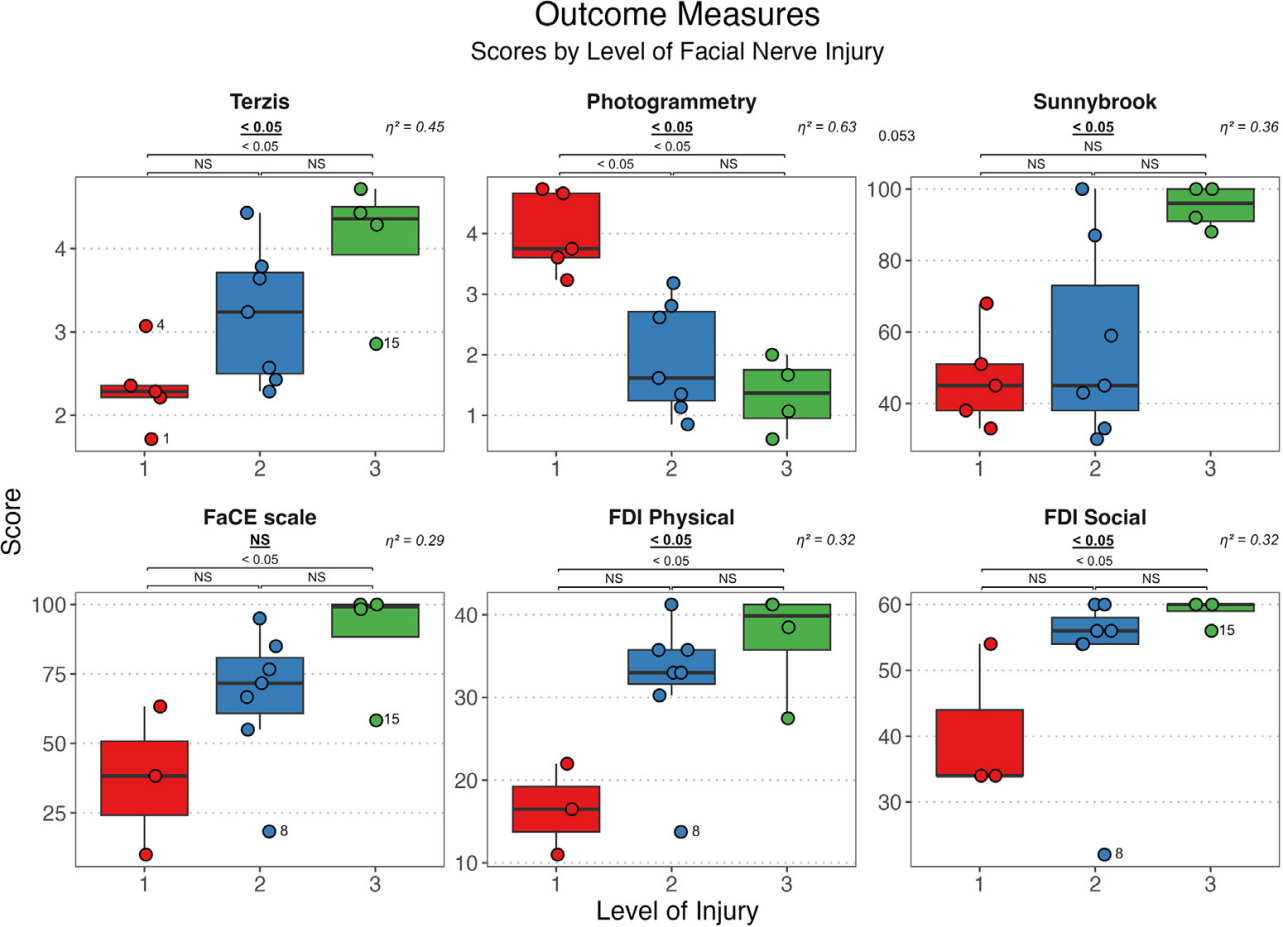
Boxplots of outcome variable scores based on the level of facial nerve injury, with each score marked by a circle. Note that the results of the photogrammetry analysis represent the mean “asymmetry between facial halves;” therefore, lower scores are better, indicating less asymmetry. For all other scores, higher scores are better. p Values for the main statistical test (Kruskal-Wallis) are shown directly below the name of each outcome parameter (underscored and bold), indicating general significance. P values from the post-hoc subgroup analyses are presented below (comparing the differences between the levels of injury: 1–2, 1–3, 2v3). The effect sizes (η2) are shown above each boxplot. Outliers are marked with their corresponding patient ID next to the circle.

Surgical outcomes were evaluated according to the level of the facial nerve injury. All results and statistical analyses, including the p values and effect sizes, are shown in Figure 2. As shown in Figure 2, Level 3 injuries yielded the best outcomes, followed sequentially by Level 2 and Level 1 injuries. This trend was consistent across all outcome parameters, with statistically significant differences in all variables except for the FaCE scale, which yielded a *p*-value of 0.055. Post-hoc analyses were performed across all variables, including the FaCE scale, because of its borderline significance. This revealed that the differences were primarily between Levels 1 and 3, which were statistically significant for all outcome parameters except Sunnybrook, which had a p value of 0.053. The difference between Levels 1 and 2 was significant in the photogrammetry analysis (p = 0.029). Notably, the effect sizes for all outcome parameters were large, with all η^2^ values exceeding 0.29, indicating a substantial impact of the level of injury on variability within all outcome parameters.

## Discussion

This small retrospective study showed that surgical outcomes differ significantly depending on the location of facial nerve injury in patients undergoing MMN reconstruction. This was demonstrated using subjective, objective, and quality-of-life outcome measures. The simple three-level anatomical classification system could divide patients into groups. Results showed that distal injuries (Level 3) had better outcomes than intraparotid injuries (Level 2), while results for more proximal injuries (Level 1) were the worst (Figure 2).

The differences in outcomes between the three levels are inherently multifactorial, likely resulting from variations in the extent and complexity of the nerve injuries across the levels. Factors such as varying nerve gap lengths, the complexity of reconstruction, and available reinnervation strategies, likely contribute to these disparities. Moreover, proximal injuries tend to entail more intricate postoperative rehabilitation regimens than isolated distal MMN injuries, potentially making them more challenging to rehabilitate. Using simple anatomical definitions, the classification system effectively captured the multifactorial factors contributing to the differences between injury levels, offering a straightforward method for stratifying patients. This suggests its potential utility in comparing surgical outcomes, enabling the grouping of patients with similar injuries to ensure comparability in characteristics and facilitate more meaningful evaluations in future research. The results are discussed below based on the treatment options available at the different injury levels.

### Treatment options for level 1 and level 2 injuries

In facial nerve injuries corresponding to Levels 1 and 2, nerve transfers are often chosen for MMN reconstruction.^23^ For the proximal Level 1 injuries, this choice is dictated by the unavailability of the proximal facial nerve stump, whereas in Level 2 injuries, despite the proximal facial nerve being available, it is generally reserved for reinnervation of the prioritized periorbital region. Consequently, MMN repair with or without an interposition nerve graft, which can yield superior outcomes, is not possible in either of these situations, necessitating nerve transfer. Selective direct nerve transfers provide a reliable and prompt supply of axons to the motor end plates, which is beneficial in cases where quick reanimation is desired and to prevent muscle atrophy and synkinesis.^8^^,^^13^^,^^24^^,^^25^

**Figure 3:**
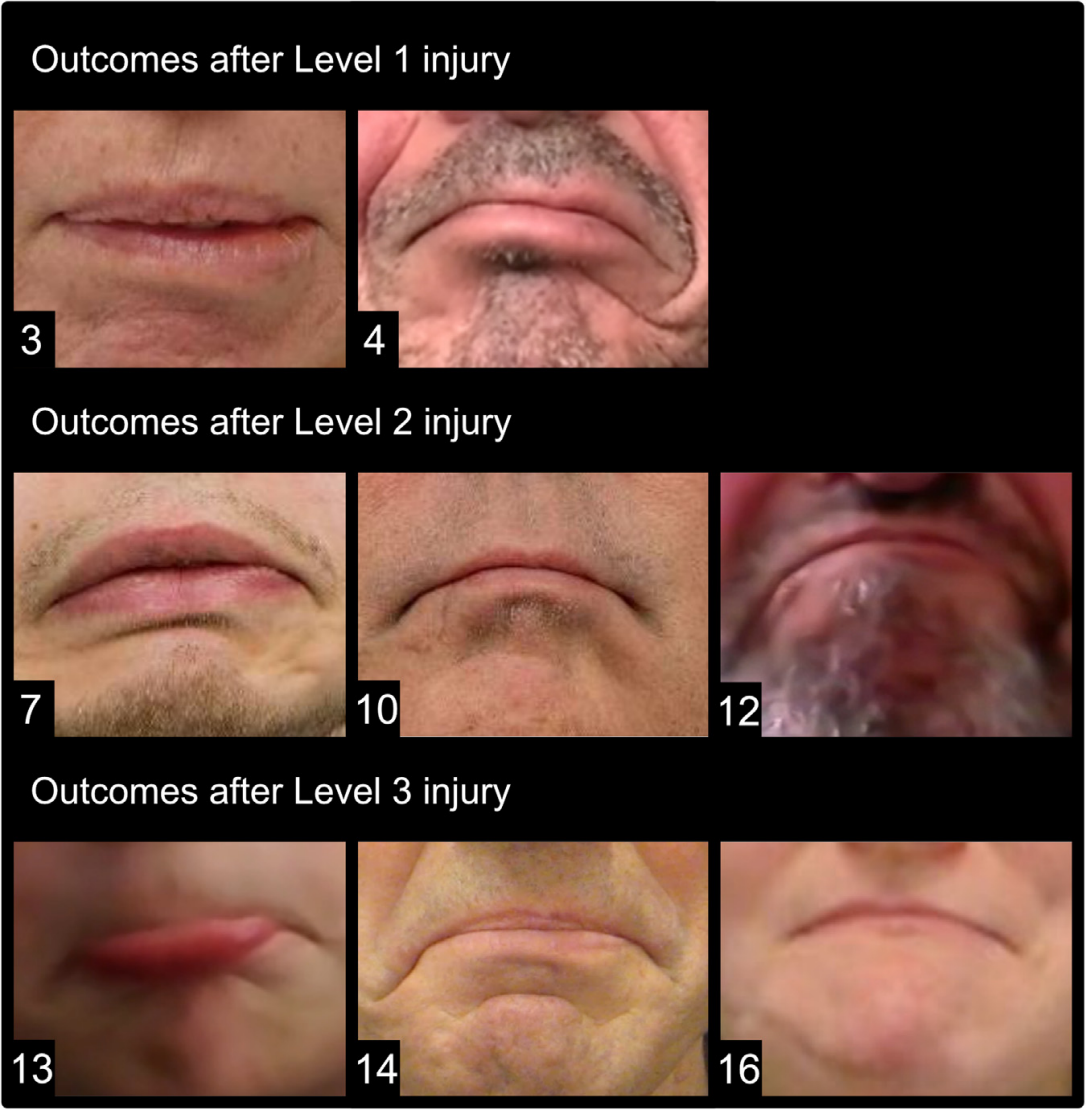
Outcomes after marginal mandibular nerve (MMN) reconstruction across the 3 injury levels. Each patient was asked to depress their lower lip to form an unhappy expression. Each photograph is marked with the corresponding patient ID from Table 2.

In most Level 2 injuries, large facial nerve resections lead to a generous surgical field, where the cervical branch has often been sacrificed. In such cases, the ansa cervicalis nerve is readily accessible and should therefore be considered. It can provide tone and balance to the lower lip^26^; in certain cases, a powerful contraction may also be achieved, as seen in the current study (Figure 3, Patient 7). The hypoglossal nerve, on the other hand, can be used to avoid the need for a longer neck incision in cases where a neck dissection is not required.^13^ A direct nerve transfer to the MMN is preferred over an indirect nerve transfer with an interposition nerve graft, given the benefits of a vascularized nerve, faster reinnervation time, and lower risk of axonal loss across the single, distal coaptation.^27^, ^28^, ^29^, ^30^, ^31^, ^32^ However, cross-facial nerve grafts from the contralateral cervical branch provide an appropriate alternative when ipsilateral donors are not available.^33^^,^^34^

In the current study, most Level 1 and 2 injuries were caused by tumors or tumor resections (11/12). In these cases, 9 reconstructions were performed using nerve transfers with either the ansa cervicalis nerve (n = 4) or the hypoglossal nerve (n = 5). Compared with nerve repairs, these transfers require more physiotherapy to reliably activate the correct muscles and are less likely to produce spontaneous movements. Moreover, the intracranial nature of Level 1 injuries often results in delays between nerve injury and nerve repair, whereas repairs for Level 2 and 3 injuries typically occur within a significantly shorter timeframe, often during the same surgical procedure. This, together with the high rate of involuntary contraction of the mentalis muscle, might explain the poorer results in these groups, particularly in older patients. Furthermore, the more extensive facial nerve involvement in Levels 1 and 2 might also lead to less focus on rehabilitation of the lower lip, as both the patient and healthcare providers might prioritize rehabilitation of the upper and middle face.

Three patients in this group stood out from the rest. In 1 patient with a Level 1 injury caused by vestibular schwannoma, with the resection leaving no nerve donors on the ipsilateral side (Patient 1 in Table 2), a cross-face nerve graft from the contralateral cervical branch was used. Another patient with a Level 1 injury due to right-sided structural compression of the facial nerve by the vertebral artery (Patient 4 in Table 2) underwent hypoglossal-MMN nerve transfer. Unlike in patients with tumors, no cheek incisions were necessary, and the hypoglossal transfer required minimal additional incisions for nerve harvesting. The outcome at the 18-month follow-up showed excellent lower lip function (Figure 3, Patient 4). However, the quality-of-life questionnaires showed poor functional and psychosocial outcomes, mainly because the patient had difficulty achieving a spontaneous smile by biting through the masseter transfer to the upper lip, despite excellent symmetrical smiling when biting. In 1 patient with a Level 2 injury and soft tissue defect following parotidectomy (Patient 12 in Table 2), the proximal MMN stump was available, enabling nerve repair. However, a nerve gap of 7.5 cm was present. The patient received a chimeric anterolateral thigh flap using the motor nerve branch of the vastus lateralis muscle to bridge the gap, with excellent results after 14 months (Figure 3, Patient 12).

**Table 2:** Table with patient data.

Table of the full patient cohort														
Patient ID	Age (years)	Gender	Etiology	Level of injury	Paralysis side	Surgical method	Follow-up length	Clavien-Dindo^a^	FDI physical	FDI social	Face scale	Sunnybrook	Photogrammetry (mean)	Terzis' Lower Lip Grading Scale score (mean)
1	23	F	Tumor	1	Left	Cervical branch (cross-face)	57	3	NA	NA	NA	68	4.7	1.7
2	25	F	Tumor	1	Left	Hypoglossal	12	0	16	34	63	45	3.8	2.2
3	51	F	Tumor	1	Left	Hypoglossal	32	3	22	54	38	33	3.2	2.3
4	54	M	Structural	1	Right	Hypoglossal	26	0	11	34	10	51	3.6	3.1
5	66	F	Tumor	1	Right	Hypoglossal	40	0	NA	NA	NA	38	4.7	2.4
6	34	M	Tumor	2	Left	Ansa cervicalis	18	3	33	60	77	33	1.3	2.3
7	27	M	Tumor	2	Left	Ansa cervicalis	18	0	30	54	55	43	2.6	3.2
8	65	F	Tumor	2	Right	Ansa cervicalis	12	0	14	22	18	45	1.1	2.6
9	71	M	Tumor	2	Left	Ansa cervicalis	35	2	36	56	85	59	0.9	2.4
10	55	M	Tumor	2	Right	Hypoglossal	28	0	33	56	72	30	3.2	3.8
11	61	M	Tumor	2	Left	Hypoglossal	47	0	41	60	95	87	2.8	3.6
12	56	M	Tumor	2	Right	Nerve graft	56	3	36	54	67	100	1.6	4.4
13	6	M	Trauma	3	Left	Cervical branch	14	0	41	60	98	100	0.6	4.7
14	66	M	Tumor	3	Left	Cervical Branch	30	0	38	60	100	100	1.1	4.3
15	64	F	Tumor	3	Right	Cervical branch (cross-face)	55	0	28	56	58	88	1.7	2.9
16	20	F	Trauma	3	Right	Direct nerve repair	18	2	41	60	100	92	2.0	4.4

FDI, Facial disability index.

a Regarding the Clavien-Dindo score, 5 patients had complications classified according to the Clavien-Dindo classification. Three patients had mild complications: 2 had postoperative infections treated with oral antibiotics (Patients 9 and 16), and 1 had a level 3 complication resulting from a hematoma and recurring eye infections due to corneal abrasion and a closure defect (Patient 3). Three patients had grade 3 complications: 1 from an ALT harvest site rupture (Patient 6), 1 from radiation-induced osteolysis resulting in a mandibular fracture (Patient 12), and 1 from a gracilis flap failure and extraction (Patient 1).

### Treatment options for level 3 injuries

In distal Level 3 injuries, when the proximal facial nerve stump is available, a reasonable attempt at tension-free direct coaptation is recommended and yields the most favorable results.^35^ Similar to nerve transfers, direct nerve repair is favored over repair, which utilizes an interposition nerve graft.^27^, ^28^, ^29^, ^30^, ^31^, ^32^ However, alternatives are required for Level 3 injuries with long nerve gaps. Notably, direct nerve transfer might be more advantageous than nerve repair using grafts in select cases, particularly if the donor nerve exhibits spontaneous and/or synergistic action with the MMN, such as the ipsilateral cervical branch of the facial nerve.^36^ The benefits include expedited nerve recovery and a minimized risk of axon loss due to a single distal anastomosis, leveraging the nerve's synergistic function and proximity to the MMN. Furthermore, donor-site morbidity resulting from distal nerve graft harvesting is mitigated.^24^^,^^36^ However, nerve grafting is required if the ipsilateral cervical branch is unavailable. In a setting where extensive scarring of the wound bed (acutely present or anticipated from radiotherapy) could compromise the survival of a non-vascularized nerve graft or a combined nerve gap and a soft tissue defect, a vascularized nerve graft is recommended, and even more so for long nerve gaps.^37^, ^38^, ^39^, ^40^, ^41^

In the current study, the 4 patients with Level 3 injuries (treated using either direct MMN repair or cervical nerve transfer) had the best postoperative outcomes. Direct repair or transfer using the ipsilateral cervical branch generates rapid and reliable results when the proximal facial nerve is accessible. Two patients presented with Level 3 injuries and MMN paralysis following trauma: a 6-year-old boy with a dog bite on the lower left face and a 20-year-old woman involved in a motor vehicle accident with a glass shard cutting into the right cheek (Patients 13 and 16). The boy had a 1 cm nerve gap, precluding direct nerve repair. Nerve transfer using the ipsilateral cervical nerve branch was performed, and the 3-month postoperative follow-up demonstrated normalized lower lip function (Figure 3, patient 13). The woman underwent direct nerve repair with excellent outcomes at the 18-month follow-up (Figure 3, Patient 16). The remaining 2 patients (Patients 14 and 15) underwent cervical branch transfers to the MMN following the excision of local tumors, where 1 patient (Patient 15) received a cross-face nerve graft from the contralateral side. Both the patients achieved good results (Table 2 and Figure 3).

**Figure 4:**
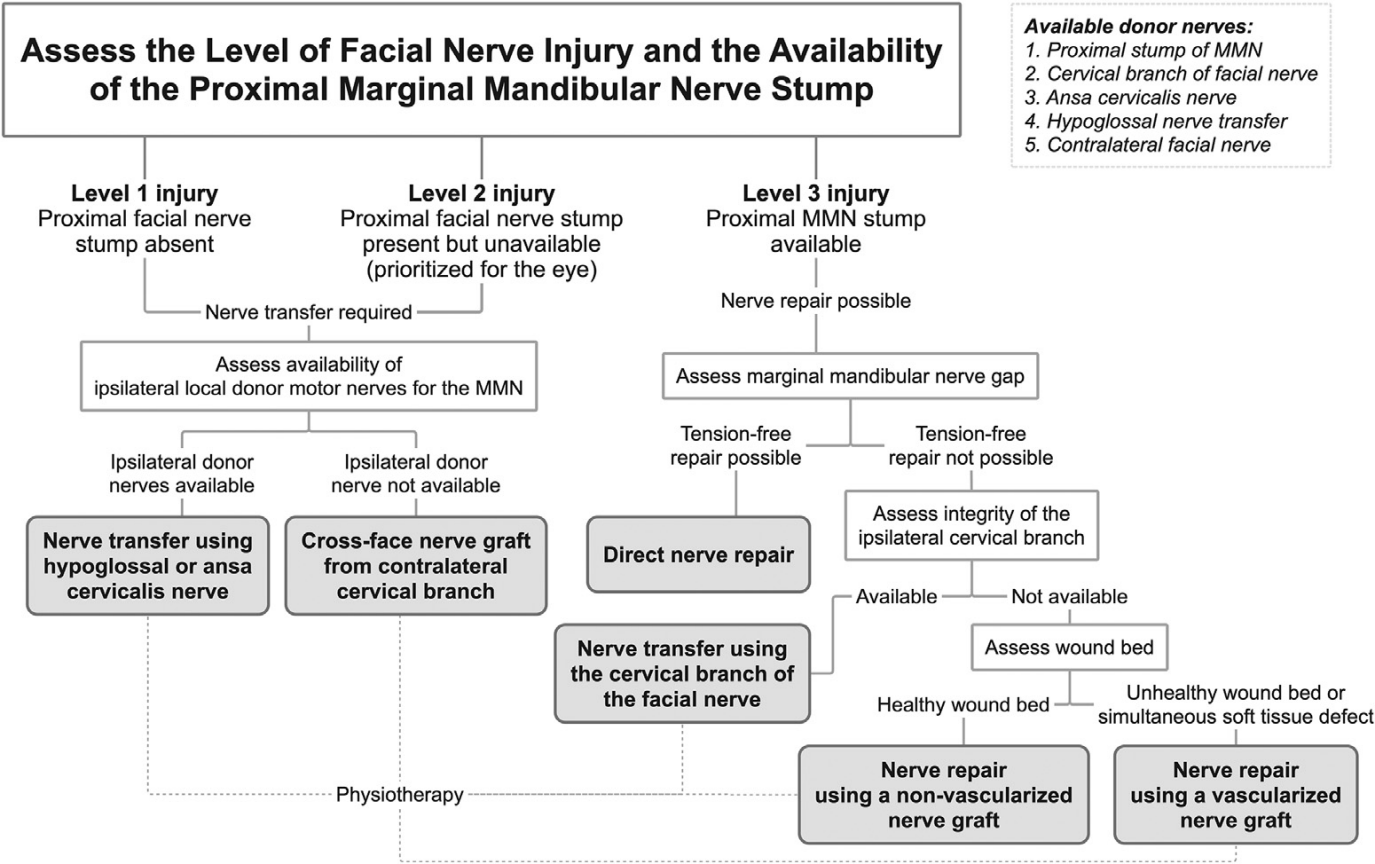
Proposed management algorithm for the reconstruction of the marginal mandibular branch of the facial nerve. Start by assessing the level of facial nerve injury and the availability of the proximal marginal mandibular nerve (MMN) stump to determine the possibility of direct nerve repair. Go through the algorithm to determine the available options for MMN reconstruction. Available donor nerves are listed in the top-right corner.

Finally, regardless of the level of injury or the chosen surgical approach, postoperative physical therapy should be regarded as an essential component of the treatment plan.^42^^,^^43^ Figure 4 presents an algorithm summarizing the above.

### Limitations

The primary limitation of this study was its small sample size, with certain surgical methods and injury levels represented by only a few patients. This precluded the use of standard ANOVA tests, which are more sensitive than the relatively conservative Kruskal-Wallis test. Despite this, the study still maintained power due to the substantial differences between injury levels. TThe large effect sizes across all outcome parameters further indicate the significant impact of injury level on the variability within the scores. In this study, all effect size scores exceeded 0.29, underscoring the clinical significance of injury location in surgical outcomes. However, the limited cohort size prevented multivariate analysis of the individual factors influencing the differences in outcomes across the levels. This finding warrants further investigation in future studies. Furthermore, interesting factors such as nerve gap length were not available in the retrospective material and could not be included in the analysis. In future studies, the classification system will require further validation to corroborate the observed outcomes in larger cohorts. In the present study, the classification system was applied to patients who underwent MMN reconstruction. In the future, its application to a broad facial paralysis cohort should be explored. The strengths of this study include its multifaceted outcome measures, providing a broad view of surgical outcomes using subjective, objective, and quality-of-life parameters.

## Conclusion

Reconstruction of the marginal mandibular nerve (MMN) is essential for achieving optimal outcomes in patients with facial paralysis. However, the highly heterogeneous nature of the MMN injury population, encompassing both extensive proximal facial nerve injuries and isolated distal MMN injuries, complicates meaningful comparisons of surgical outcomes. This small retrospective study demonstrated significant variability in outcomes depending on the location of facial nerve injury in patients undergoing MMN reconstruction. The proposed three-level classification system effectively stratified patients, revealing better outcomes in distal injuries (Level 3) compared to intraparotid injuries (Level 2), with the least favorable results observed in proximal injuries (Level 1). These findings underscore the importance of considering injury location when comparing surgical results. The classification system offers a practical framework for grouping patients according to their anatomical injury location, facilitating more meaningful and standardized comparisons of surgical outcomes among patients with similar characteristics and treatment protocols.

## Conflicts of interest

None declared.
